# Gene Expression Meta-Analysis of Potential Shared and Unique Pathways between Autoimmune Diseases under Anti-TNFα Therapy

**DOI:** 10.3390/genes13050776

**Published:** 2022-04-27

**Authors:** Charalabos Antonatos, Mariza Panoutsopoulou, Georgios K. Georgakilas, Evangelos Evangelou, Yiannis Vasilopoulos

**Affiliations:** 1 Laboratory of Genetics, Section of Genetics, Cell Biology and Development, Department of Biology, University of Patras, 26504 Patras, Greece; charisantonatos@gmail.com (C.A.); marizapanoutsopoulou@gmail.com (M.P.); ggeorgakila@upnet.gr (G.K.G.); 2 Laboratory of Hygiene and Epidemiology, Department of Clinical and Laboratory Research, Faculty of Medicine, University of Thessaly, 38334 Volos, Greece; 3 Department of Hygiene and Epidemiology, Medical School, University of Ioannina, 45110 Ioannina, Greece; vangelis@uoi.gr; 4 Department of Biomedical Research, Institute of Molecular Biology and Biotechnology, Foundation for Research and Technology-Hellas, 45510 Ioannina, Greece; 5 Department of Epidemiology & Biostatistics, MRC Centre for Environment and Health, Imperial College London, London W2 1PG, UK

**Keywords:** autoimmune, anti-TNFα, gene expression, meta-analysis, pharmacogenomics

## Abstract

While anti-TNFα has been established as an effective therapeutic approach for several autoimmune diseases, results from clinical trials have uncovered heterogeneous patients’ response to therapy. Here, we conducted a meta-analysis on the publicly available gene expression cDNA microarray datasets that examine the differential expression observed in response to anti-TNFα therapy with psoriasis (PsO), inflammatory bowel disease (IBD) and rheumatoid arthritis (RA). Five disease-specific meta-analyses and a single combined random-effects meta-analysis were performed through the restricted maximum likelihood method. Gene Ontology and Reactome Pathways enrichment analyses were conducted, while interactions between differentially expressed genes (DEGs) were determined with the STRING database. Four IBD, three PsO and two RA datasets were identified and included in our analyses through our search criteria. Disease-specific meta-analyses detected distinct pro-inflammatory down-regulated DEGs for each disease, while pathway analyses identified common inflammatory patterns involved in the pathogenesis of each disease. Combined meta-analyses further revealed DEGs that participate in anti-inflammatory pathways, namely IL-10 signaling. Our analyses provide the framework for a transcriptomic approach in response to anti-TNFα therapy in the above diseases. Elucidation of the complex interactions involved in such multifactorial phenotypes could identify key molecular targets implicated in the pathogenesis of IBD, PsO and RA.

## 1. Introduction

Autoimmune diseases consist of a large group of multifactorial, chronic inflammatory diseases caused by the complex interaction between genetic and environmental factors [1], leading to severe pathological symptoms that detriment a patient’s quality of life [2]. The extended tissue damage and severe clinical outcomes of autoimmune diseases, accompanied by high, direct and indirect annual costs to the health care system [3] emphasize the necessity for an efficient therapy. The development of innovative treatments, and especially protein-based drugs (biological agents), has emerged as a revolutionary method for treating multiple autoimmune diseases that aims to control the overactive immune response and alleviate the inflammatory signs [4].

Increasing knowledge regarding the pathophysiology of specific chronic inflammatory diseases and, specifically, inflammatory bowel disease (IBD) (consisting of Crohn’s disease (CD) and ulcerative colitis (UC)), psoriasis (PsO) and rheumatoid arthritis (RA), has provided insights into the underlying mechanisms that maintain the inflammation, such as the mechanisms promoted by Tumor Necrosis Factor Alpha (TNFα) [5]. Such inflammation occurs in IBD, for example, due to the combination of several environmental risk factors and genetic predisposition that are heavily associated with the dysbiosis of gut microbiota, such as those promoted by the *NOD2* gene which regulates the host’s immune response through the recognition of bacterial derivatives [6]. The resulting inflammation in the gastrointestinal tract, caused mainly by TNFα and interleukin (IL) 6, leads to extensive damage in the intestinal epithelium and endothelium, as well as alterations in the extracellular matrix [7].

In the case of PsO, the activation of antigen-presenting immune cells by several epidermal autoantigens produced by keratinocytes, induces the secretion of pro-inflammatory cytokines, which directly stimulate T helper (Th) cells [8]. Consequently, Th cells enhance keratinocyte hyperproliferation, mainly through the secretion of TNFα and IL17A, while keratinocytes maintain the chronic inflammatory cycle through the production of multiple chemokines [9].

In the framework of RA, the disease progression is divided into several distinct clinical stages, determined by the interactions between genetic and environmental risk factors [10]. The well-studied TNFα signaling pathway in the synovial fibroblasts precipitates their aggressive hyperplastic and invasive phenotype, causing major damage to the cartilage [11].

The anti-TNFα biological agents are considered an ideal therapeutic approach in the above diseases, given the predominant role of TNFα in their pathogenesis. Five anti-TNFα agents have been developed and are widely used as therapeutic agents, including infliximab (IFX), adalimumab (ADA), etanercept (ETC), certolizumab-pegol (CEP) and golimumab (GOL) [12,13]. Despite the efficacy of anti-TNFα agents, several clinical trials have shown that 20–40% of patients do not respond to treatment (primary non-responders), while almost half of the benefited patients will lose these clinical benefits within the first year of treatment (secondary loss of response) [14]. This observation is attributed, among other factors, to the patient’s genetic background [15]. Therefore, multiple studies have evaluated the patients’ response to anti-TNFα agents according to their genetic background, supporting the notion of the genetic variation significance in their response to treatment [16,17,18]. This has been confirmed through meta-analyses on large cohorts of patients suffering from PsO, IBD and RA [19]. However, this approach is limited by the fact that is based on the analysis of selected genetic loci, yielding data that could only partially attribute the clinical heterogeneity to anti-TNFα therapy response.

During the last two decades, high-throughput technologies and -omics platforms have been used to elucidate the complex interactions in multifactorial phenotypes [20], assessing a wide variety of conditions. Transcriptomics focus on the quantification of gene expression, either with the usage of cDNA microarrays, or with RNA-seq [21]. In this context, gene expression profiling through cDNA microarrays has been implemented in the investigation of the diverse response to anti-TNFα therapy in IBD [22,23,24], PsO [25,26,27] and RA [28,29]. Nevertheless, the reported findings of such high-throughput experiments are rarely consistent and reproducible, due to the variable analytical methodologies performed and platforms used in each study. Additionally, the limited number of included patients aggravates the existing heterogeneity [30,31].

In this study, we systematically screened all available cDNA microarray datasets between responders and non-responders to anti-TNFα therapy, in patients with IBD, PsO and RA, and conducted a random effects meta-analysis, aiming to identify unique and shared genes and pathways that could be potentially related to the phenotypically complex response to therapy.

## 2. Materials and Methods

### 2.1. Identification of Eligible Datasets and Inclusion/Exclusion Criteria

**Figure 1:**
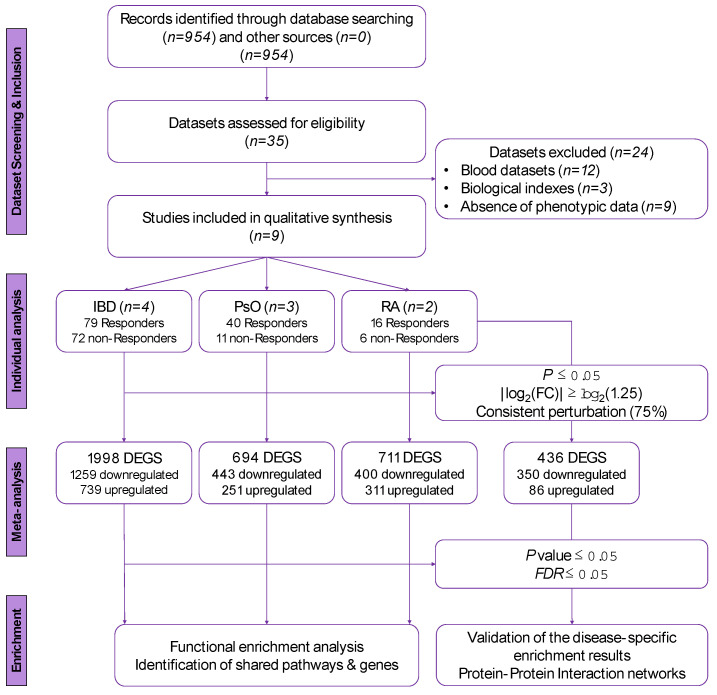
Schematic overview of our study.

A schematic overview of our research workflow is presented in Figure 1. We considered all original experimental datasets that examined changes in gene expression between responders and non-responders to anti-TNFα therapy, in patients with IBD, PsO and RA. We systematically screened publicly available datasets on Gene Expression Omnibus (GEO) from inception on the 10 November 2020 [32]. Keywords and terms added in our search strategy included “psoriasis”, “Crohn’s disease”, “ulcerative colitis”, “rheumatoid arthritis” and similar search terms, filtering for “Homo sapiens”. The gene expression microarray datasets used in our analyses were filtered to include adult human case-control studies on anti-TNFα response in the diseases under study and to correspond to samples collected from inflamed biopsies (either skin, intestinal mucosa or synovial, respectively). Datasets were excluded if they referred to blood samples, assessed response to therapy using biological indexes rather than clinical score indexes, and did not provide sufficient phenotypic data about response to therapy for each patient, either through the data submitted in the GEO platform files or through full-text mining.

### 2.2. Dataset Pre-Processing

Each raw dataset was separately downloaded from GEO using GEOquery [33]. For raw datasets that did not contain the necessary phenotypic data (metadata), the latter were downloaded and integrated into the raw ExpressionSet via the corresponding processed data. Probe expression values were quantile normalized, log_2_ transformed and annotated with the R [34] Bioconductor v. 3.14 [35] annotate v. 1.72.0 package [36]. Multiple probesets mapping to the same transcript were summarized and studied at a single gene level. Prior to Differential Gene Expression Analysis (DGEA), samples that were not relevant to our analysis (e.g., non-lesional skin biopsies) were excluded. DGEA was performed with the limma v. 3.50. 1 package [37], a well-established linear model for microarray statistical analyses. The analysis was not adjusted for covariates and the clinical characteristics of each patient due to the unavailability of individual level data in most datasets.

### 2.3. Disease-Specific and Combined Meta-Analyses

We applied the Random Effects Model (REM) approach through the restricted maximum likelihood method (RELM), implemented in the MetaVolcanoR v1.8.0 package [38], where dataset-specific gene fold changes (FC) and their representative confidence intervals (CIs) are summarized. We performed a total of six meta-analyses: three for each disease (PsO, IBD, RA), two for Crohn’s disease and ulcerative colitis and a single combined meta-analysis on all datasets. Genes derived from each meta-analysis were ranked according to the TopConfects [39] approach, implemented in the MetaVolcanoR [39] package. The top 1% of the most consistently perturbed genes in the included datasets were highlighted, while maintaining a 5% False Discovery Rate (FDR). Genes considered differentially expressed (DEGs) were those with *p* ≤ 0.05, |log_2_(FC)| ≥ log_2_(1.25) and perturbed in at least 75% of the included datasets. Visualization of DEGs was performed with the EnhancedVolcano v. 1.12.0 R package [40].

### 2.4. Over-Representation Analysis

Over-representation analysis (ORA) was performed using Gene Ontology (GO) [41] and Reactome Pathways [42] with the R clusterProfiler v. 4.2.2. package [43] for both up- and down-regulated genes in all four performed meta-analyses using the default parameters. Outputs of the GO ORA were further analyzed and reduced to single representative terms to reduce the redundancy of the GO child terms. Pairwise similarities were subsequently calculated for the biological processes (BPs) of each down-regulated gene set through Wang’s method based on the topology of the GO directed acyclic graphs (DAGs) [44]. To characterize the shared enriched functional profiles in our meta-analyses and identify key shared genes between the three diseases under the same treatment, we created gene clusters of up- and down-regulated genes from each disease and aggregated the results into a single object [43]. For each enriched pathway, the *p* value was calculated with the hypergeometric model and controlled for multiple comparisons with the Benjamini & Hochberg method. Enriched pathways with an adjusted *p* ≤ 0.05 were regarded as statistically significant.

### 2.5. Construction of Protein-Protein Interaction (PPI) Networks

To assess the interactions between the statistically significant up- and down-regulated genes from the combined meta-analysis, as well as the shared genes and their respective interactors identified through our ORA analyses, we incorporated the STRING v11.5 database [45] with a direct (physical) interaction score of 0.7 and visualized the derived networks with Cytoscape v3.9 [46].

## 3. Results

### 3.1. Included Datasets

A total of nine microarray datasets were identified in the literature search based on our prespecified criteria: four datasets referred to IBD [22,23,24], three to PsO [25,26,27] and two to RA [28,29]. IBD datasets accumulated a total of 151 patients (79 responders/72 non-responders), PsO included 51 patients (40 responders/11 non-responders) whereas RA datasets had 22 patients (16 responders/6 non-responders). Four experimental datasets were performed using the Affymetrix Human Genome U133 Plus 2.0 Array (GPL570) whereas the rest used various other platforms (Table 1). The clinical definition of response to therapy was evenly assessed in the PsO and RA datasets; PsO datasets characterized responders as those with a greater or equal than 75% improvement in the psoriasis area and severity index (PASI75) score [25,26,27], while RA responders were assessed according to the European League Against Rheumatism (EULAR) criteria [28,29,47]. As far as IBD was concerned, three datasets (GSE16879, GSE92415, GSE23597) assessed patients with ulcerative colitis (UC) as responders to anti-TNFα therapy with the Mayo scores, where a decrease of Mayo subscores of at least three points by 30%, with the exception of Arijs et al. where a decrease in Mayo endoscopic subscores considered patients as responders [23]. Crohn’s disease (CD) patients were assessed as responders either through a decrease in the Crohn’s Disease Endoscopic Index of Severity (CDEIS) of less than five (GSE52746 [22]), or complete mucosal healing with a significant decrease in the histological score [48].

### 3.2. Differentially Expressed Genes in the Disease-Specific Meta-Analyses

Our primary goal was to identify the differentially expressed gene signatures of the anti-TNFα response to therapy in the three diseases under study, as well as identify shared genes and pathways. The IBD meta-analysis revealed a total of 1998 genes, where 1258 were down-regulated and 739 were up-regulated (Supplementary Figure S1a). Similarly, meta-analysis of the three PsO datasets resulted in 694 DEGs, 443 down-regulated genes and 251 up-regulated genes (Supplementary Figure S1b). In the RA meta-analysis, 711 DEGs were detected including 400 down-regulated and 311 up-regulated genes (Supplementary Figure S1c). The top five up- and down-regulated genes in all meta-analyses are shown in Table 2 according to the *TopConfects* [39] approach implemented in the *MetaVolcanoR* [38].

The complete list of up- and down-regulated genes is provided in Supplementary Table S1. Eight genes were consistently down-regulated in all three disease-specific meta-analyses, including CCAAT/enhancer binding protein delta (*CEBPD*) and Ficolin 1 (*FCN1*), whilst the up-regulation pattern of three genes gave the same disease-specific response to anti-TNFα therapy (Supplementary Table S1).

The CD meta-analysis yielded a total of 1798 differentially expressed genes (DEGs), where 757 were up-regulated and 1041 were down-regulated. On the other hand, the UC meta-analysis gave us a total of 2297 DEGs, out of which 880 were up- regulated and 1417 were down-regulated (Supplementary Figure S5). A total of 1066 DEGs were shared between the UC and CD meta-analyses, with 685 being down- regulated and 381 being up-regulated. A full list of our DEGs, concerning both meta-analyses, is provided in Supplementary Table S1, while functional enrichment of the CD and UC DEGs is provided in our Supplementary File S1 and Supplementary Figures S6–S8.

### 3.3. Functional Enrichment Analysis of the Disease-Specific DEGs

The over-representation analysis (ORA) was performed on each disease-specific DEG set to identify the associated biological pathways and processes. ORA was initially based on the Reactome pathways database [41] and subsequently compared to the gene ontology terms [40].

**Figure 2:**
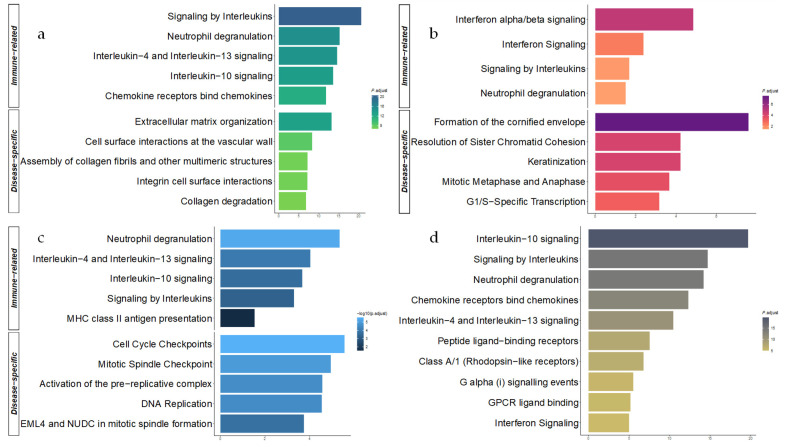
Enriched Reactome pathways of down-regulated genes in (**a**) IBD, (**b**) PsO, (**c**) RA and (**d**) Combined meta-analysis. For IBD, PsO and RA, pathways were categorized as ‘immune-related’ and ‘disease-specific’ based on biological relevance, as well as their adjusted *p*.

**Figure 3:**
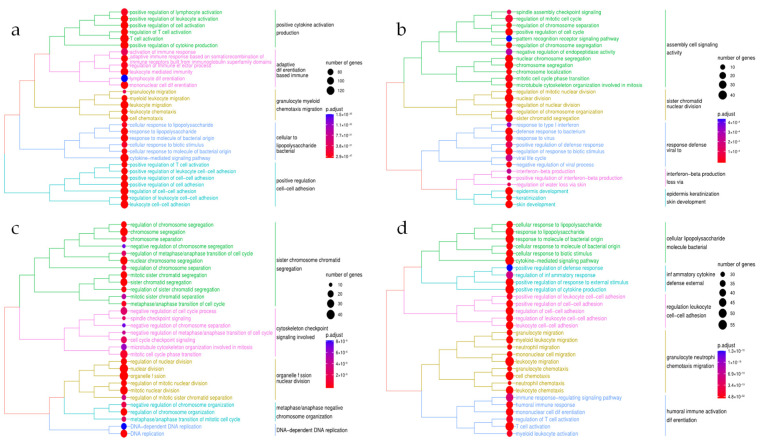
Semantic similarity analysis of the simplified biological processes concerning the down-regulated genes in (**a**) IBD, (**b**) PsO, (**c**) RA and (**d**) Combined meta-analysis.

A total of 122 Reactome pathways were enriched for the down-regulated IBD genes with numerous immune-related terms, some of which include interleukin (IL) pathways (e.g., ‘interleukin-4 and interleukin-13 signaling’ (39/108 genes) and ‘interleukin-10 signaling’ (25/47 genes)), ‘neutrophil degranulation’ (93/480 genes) as well as G-protein coupled receptor (GPCR) pathways with ‘G alpha (i) signaling events’ (53/318 genes) and ‘class A/1 (rhodopsin-like receptor)’ (57/335 genes), for instance (Figure 2a). Far less Reactome terms were over-represented concerning the up-regulated IBD genes, reaching a total of 16 distinct pathways. These terms referred to the ‘metabolic pathways of fatty acids’ (21/177 genes), ‘citric acid cycle (TCA cycle)’ (6/22 genes) as well as ‘biological oxidation’ (24/222 genes) and ‘mitochondrial beta-oxidation of saturated fatty acids’ (5/11 genes) (Supplementary Table S2). By conducting the Gene Ontology ORA, we identified more than 1000 distinct biological process (BP) terms concerning the down-regulated IBD genes, which were subsequently reduced to 492 non-redundant BPs, 64 molecular function (MF) and 35 cellular component (CC) terms (Supplementary Table S3). Semantic similarity analysis confirmed the significant immunological background of the response to anti-TNFα therapy in patients with IBD, through the identification of leukocyte-associated terms and adaptive immunity, characterizing a distinct pattern of cell responses to molecules of bacterial origin (Figure 3a). Up-regulated IBD genes were significantly enriched for 232 BP, 12 MF and 28 CC terms, while 110 BP terms remained after the reduction to single representative terms. Metabolic processes such as pathways included in the biosynthesis of carbohydrates, breakdown of organic acids and lipids were identified through the ORA (Supplementary Table S3).

Enriched Reactome pathways (*n* = 35) in down-regulated PsO genes revealed a distinct functional enrichment comprising pathways associated with keratinocytes, such as the ‘formation of the cornified envelope’ (21/130 genes) and ‘keratinization’ (21/214 genes), in conjunction with numerous cell cycle and mitosis-related pathways, namely ‘mitotic metaphase and anaphase’ (21/237 genes), ‘mitotic G1 phase and G1/S transition’ (14/149 genes) and ‘regulation of mitotic cell cycle’ (10/88 genes). Additionally, PsO down-regulated genes were enriched with immune signaling pathways, namely ‘interferon alpha/beta’ (13/70 genes) and ‘interleukin-mediated signaling’ (25/462 genes; Figure 2b). Consistent with the functional analysis performed in IBD, pathways associated with the up-regulated PsO genes were significantly less (*n* = 15) and mainly related to proteoglycans due to the presence of *CSPG4*, *GPC2*, *GPC6*, *BGN* and *HSPG2* genes (Supplementary Table S2). GO ORA on the down-regulated PsO genes revealed a total of 257 BP, 16 MF and 39 CC GO terms, reduced to 107 BP, 10 MF and 16 CC terms when considering the overlapping child GO terms (Supplementary Table S3). The semantic similarity analysis of the GO BP terms of the down-regulated PsO genes further highlighted the importance of the cell cycle and mitosis, in the skin biopsies derived from the PsO responders, in the response to anti-TNFα therapy; terms including chromosome segregation and nuclear division were abundant, along with skin-related terms (e.g., keratinization and skin developmen t), whilst immune-related pathways mainly referred to interferon signaling and response to viral infection, as shown in Figure 3b. On the other hand, the 13, non-redundant BP terms associated with the up-regulated PsO genes were significantly enriched for the development and differentiation of muscle and connective tissue (Supplementary Table S3). Notably, BP terms referring to the morphogenesis of the epithelial tissue as well as the regulation of epithelial cell proliferation and migration were not statistically enriched.

Similarly, the ORA of down-regulated RA genes detected multiple cell-cycle-associated pathways including ‘cell cycle checkpoints’ (28/294 genes), ‘DNA replication’ (16/128 genes) and ‘G1/S transition’ (14/131 genes), while immune-related pathways such as ‘neutrophil degranulation’ (36/480 genes), ‘interleukin-4 and interleukin-13 signaling’ (14/126 genes) and ‘signaling by interleukins’ (29/462 genes) were significantly enriched (Figure 2c). The up-regulated RA genes, however, were not significantly over-represented for any Reactome pathway (Supplementary Table S2). Considering the GO over-representation analysis on the RA DEGs, 296 BP, 7 MF and 51 CC terms were statistically enriched; non-redundant GO terms were consequently reduced to 107 BP, 7 MF and 17 CC terms (Supplementary Table S3). Out of the 107 non-redundant BP terms, semantic similarity analysis revealed, comparably to the Reactome pathways ORA, numerous cell cycle and nuclear division processes, including distinct immune terms such as the regulation of cytokine production and response to interleukin-1 (Figure 3c). Contrary to the Reactome ORA, GO analysis showed 24 BP, 3 MF and 5 CC terms concerning the up-regulated RA genes which were reduced to 20 BP, 2 MF and 5 CC representative terms. The 20 BP terms were mostly associated with metabolic processes of retinol and terpenoid, in addition to the positive regulation and activation of protein kinase B (PKB) activity (Supplementary Table S3).

Comparison of the three disease-specific down-regulated genes in the ORA in the Reactome pathways database, unveiled two common pathways; Neutrophil degranulation and signaling by interleukins, with *CEBPD* and *FCN1* being present in all disease-specific DEGs (Supplementary Table S2).

### 3.4. Differentially Expressed Genes in the Combined Meta-Analyses

Due to the disease-specific patterns noticed in the three above sub-analyses, we conducted a random effects meta-analysis on all nine datasets. A total of 2022 genes showed a consistent gene expression pattern throughout the nine included datasets (Supplementary Figure S2d). Out of those, 350 were found to be down-regulated and 86 up-regulated. The top ten up- and down-regulated DEGs are shown in Table 2, the order of which is derived from the TopConfects approach [39], while the full list of the consistently perturbated genes is provided in Supplementary Table S1. As expected, genes that were differentially expressed in all three disease-specific meta-analyses also maintained this pattern in the combined meta-analysis (Supplementary Table S1).

### 3.5. Functional Enrichment in the Combined Meta-Analysis

Forty-nine Reactome pathways were significantly enriched concerning the 350 down-regulated genes in the combined meta-analysis. Most of the enriched pathways referred to immune-related signaling events and antigen-presenting pathways, such as ‘interleukin-10 signaling’ (22/47 genes), ‘interferon alpha/beta signaling’ (12/108 genes) and multiple toll-like receptor (TLR) signaling cascades. Out of the 49 statistically enriched pathways, seven were not present in the disease-specific meta-analyses, even though they exhibited a low gene counts ratio. Out of those, six were immune-related, including ‘interleukin-6 signaling’ (3/11 genes) and ‘FcεRI-mediated Ca^2+^ mobilization’ (5/33 genes), while ‘regulated necrosis’ (7/57 genes) was also significantly enriched (Figure 2d). On the other hand, the up-regulated genes were not enriched for any Reactome pathway terms (Supplementary Table S2). Furthermore, the GO ORA identified 898 BP, 55 MF and 36 CC terms that were significantly enriched concerning the down-regulated genes, which were afterwards reduced to 256 BP, 27 MF and 20 CC ontology terms. Semantic similarity analysis on the non-redundant 256 BP terms showed numerous immune-related processes, such as the chemotactic migration of leukocytes, cytokine-meditated events, as well as regulation of the immune response through biotic stimuli (Figure 3d). In addition, 78 BP terms were enriched in up-regulated genes from the combined meta-analysis, while MF and CC terms did not give results. More than half of the BP terms were maintained as single representative terms; multiple cell differentiation processes were discovered, including stem and fat cell differentiation, whilst, as noted in every disease-specific meta-analysis, metabolic pathways were also present with steroid, fatty acid and nucleotide metabolic processes, for instance (Supplementary Table S3).

### 3.6. Protein–Protein Interaction Network

**Figure 4:**
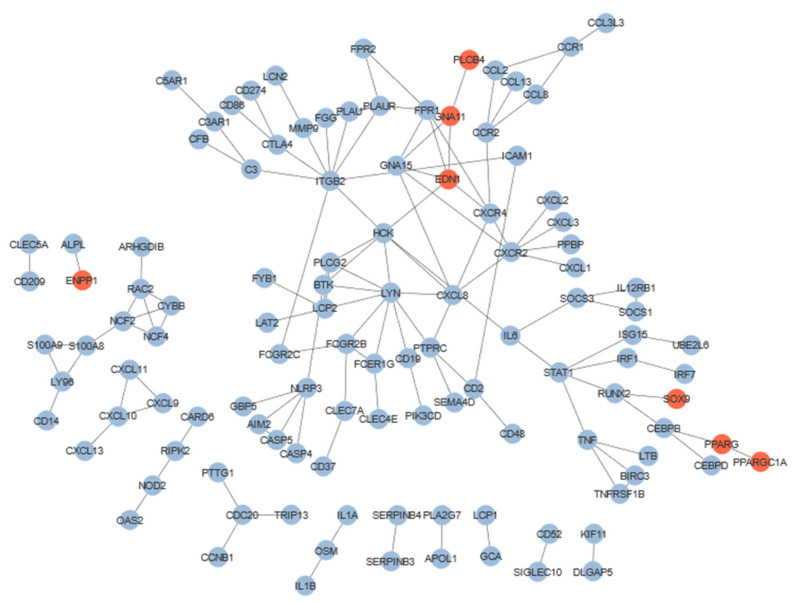
Physical protein–protein interactions between the differentially expressed genes, as derived from our combined meta-analysis (score threshold > 0.7). Blue nodes represent down- regulated and red nodes up-regulated genes.

Protein–protein interaction (PPi) networks were incorporated to elucidate the complex interactions taking place in the response to anti-TNFα therapy in the diseases under study, as well as to characterize specific patterns of the deregulated genes. DEGs of the combined meta-analysis derived a PPi network consisting of more than 400 nodes with a PPi enrichment *p* < 10^−16^. Out of those, 114 genes were noticed to be interconnected inside our network (Figure 4). Three shared down-regulated genes were found inside the sub-networks of our PPi; *GNA15*, *CEBPD* and *TRIP13*. TRIP13 interacts with CDC20 with a score of 0.961, both participating in the spindle assembly checkpoint. Furthermore, *CEBPD* and *GNA15* were identified inside our largest sub-network, as derived from our PPi analysis. CEBPD is a transcription factor that forms a heterodimer with CEBPB (PPi score = 0.991), which further regulates the transcription of IL-6, a key proinflammatory cytokine overexpressed in multiple autoimmune diseases. On the other hand, GNA15 is involved in G-protein signaling events, as seen through our enrichment analyses (Supplementary Table S2), which is further explained through its interactions with various proteins including chemotactic factors (FPR1, CXCL8) and the preproendothelin-1 (EDN1), with an interaction score >0.9 in all cases.

## 4. Discussion

In this study, we considered microarray datasets that assessed the differential gene expression observed between responders and non-responders in autoimmune diseases with anti-TNFα as their main pharmacotherapy. Four datasets studying IBD [22,23,24], three associated with PsO [25,26,27] and two with RA [28,29] were included, with samples derived from the respective inflamed tissue. Multiple methodologies have been suggested for microarray meta-analyses aiming to assess both technical and biological heterogeneity between the datasets under study [49]. Out of those, we incorporated a random effects meta-analysis approach through the restricted maximum likelihood method (RELM), aiming to identify shared and unique genes and pathways that are differentially expressed by the complex response to therapy phenotype.

Our disease-specific meta-analyses detected almost 2000 deregulated genes in the IBD datasets, while the number of differentially expressed genes (DEGs) in PsO and RA were less than half. Such a vast disparity between the observed DEGs in each disease-specific meta-analysis, compared to IBD, partially lies in the clinical subtypes of the latter, Crohn’s disease (CD) and ulcerative colitis (UC). Although CD and UC display a significant genetic risk loci overlap [50], as well as a similar stabilizing but, nevertheless, high prevalence in westernized societies [51], the distinct histological localization and inflammatory manifestation throughout the gastrointestinal epithelium underlie pathological differences between both diseases, which are reflected in the transcriptome profiling of therapy response. Nevertheless, the same vast differences were observed during our CD and UC separate meta-analyses (Supplementary File S1). This could be further attributed to the dissimilarities noticed in the responders/non-responders (R/NR) ratio included in each disease-specific meta-analysis. Specifically, the R/NR ratio in the IBD datasets was close to equal (79/72), a trend further maintained in the CD (27/21) and UC (52/51) datasets, unlike the PsO (40/11) and RA (22/6) meta-analyses. Furthermore, residual heterogeneity of the *p* values of the statistically significant IBD genes were a lot smaller (Supplementary Figure S2) in comparison with the PsO and RA heterogeneity *p* values (Supplementary Figure S2). Similar density plots were derived from the heterogeneity of the *p* values of the combined meta-analysis (Supplementary Figure S3), as well as for CD and UC (Supplementary Figure S4). Although the combined meta-analysis was expected to yield such a high heterogeneity due to the clinical and, therefore, transcriptomic discrepancies between the included datasets, the heterogeneity present in our IBD, CD and UC meta-analyses warrants consideration for further in-depth studies. Eleven genes (eight down-regulated and three up-regulated) showed a consistent deregulated expression pattern in all disease-specific analyses (Supplementary Table S1). Strikingly, six out of eight down-regulated genes participate in the regulation of cell cycle, mitotic and G protein signaling pathways; the immune-related genes consisted of CCAAT/enhancer binding protein delta (*CEBPD*) and Ficolin 1 (*FCN1*), as seen through our over-representation (ORA) analyses (Supplementary Table S2). Both genes participate in the two shared pathways between the disease-specific analyses, signaling by interleukins and neutrophil degranulation, respectively. *CEBPD* is a transcription factor involved in multiple cellular signaling events, including cell proliferation and proinflammatory pathways [52]. Specifically, *CEPBD*’s transcription is mediated from the NF-kB pathway [53] and forms stable heterodimers with CEBPB which promote the transcription of *IL6* [54]. However, several studies have assessed the role of *CEBPD* in the inflammation occurring in each disease under study with contradictory findings [55,56,57,58,59]. For example, *CEBPD*-deficient-collagen-induced arthritis mouse models had a lower RA activity compared to the wild type [56], whilst knockout of *CEBPD* in dextran-sulfate sodium (DSS)-induced colitis mice resulted in a higher susceptibility to the disease [57]. These results suggest that *CEBPD* could display a tissue-specific interaction network that could either aggravate or reduce the inflammation. In contrast, *FCN1* is part of the ficolin protein family with an extracellular pattern-recognition receptor functionality which activates the lectin complement pathway [60]. Our results concerning the down-regulation of *FCN1* during the response to anti-TNFα therapy are consistent with previous studies assessing the activity of the lectin pathway in responders with IBD [61], while -omics profiling of biopsies derived from patients with Crohn’s disease (CD) and ulcerative colitis (UC) showed a significant up-regulation of *FCN1* [62,63]. In addition, FCN1 inhibition alleviated the symptoms of multiple autoimmune diseases in mouse models, including collagen-induced RA mice, further suggesting its crucial role in the development of inflammation [64].

To identify shared pathways and gene expression perturbation among our three diseases under study, we conducted a combined meta-analysis on each separate dataset. Our results, based on our predefined criteria, displayed about a four-times greater number of down-regulated genes (350) compared to the number of up-regulated genes (86), which could be addressed in the elimination of the disease-specific DEGs, as noticed through our over-representation analyses (Figure 2d and Figure 3d). The 11 genes that were found to exhibit the same deregulation patterns in the three disease-specific meta-analyses were also unveiled to be differentially expressed in the combined meta-analysis. Interestingly, only three proteins encoded by these 11 genes were found in our physical PPi network presented in Figure 4. This could be attributed to the inherent properties of the PPi networks that enable them to only capture part of the functional aspect of canonical or deregulated gene expression. Since some of the 11 genes are encoding for transcription factors, it is expected that a gene regulatory network (GRN) approach would be highly beneficial for unveiling the importance of these genes in explaining the under-study of disease phenotypes, as well as reinforcing our understanding about the common or different biological frameworks between IBD, PsO and RA. Analyses based on GRNs have multiple advantages that stem from the multilayered functional nature of incorporated regulators and targets [65]. Nodes can represent protein-coding or non-coding genes (microRNAs, long non-coding RNAs, etc.), transcription factors and RNA binding proteins among others. The complexity of biological systems is captured by node-connecting edges in GRNs that conceptualize the spectrum of regulatory relationships between nodes (activation, repression, modulation, etc.,). Thus, GRN-based approaches can also assist in capturing the importance of genes with limited expression variance between different biological conditions, hiding behind the statistical frontline of DGE analyses. However, building robust GRN inference models typically requires the combination of datasets from multiple types of experiments, which was a key limiting factor in this study.

Such limiting factors are also inherent in the microarray technology; microarrays are based on fluorescence intensities to quantify gene expression through the hybridization of anti-sense probes to specifically target sequences. Hence, microarray technique could be characterized as a biased approach in the quantification of a sample’s transcriptome, thus restricting the identification of the above regulatory elements that are implicated in such multifactorial phenotypes. Inconsistencies noticed between the number of DEGs in each disease-specific meta-analysis is further magnified from the discrepancy of the number of patients included in each meta-analysis, where the number of patients with IBD is three-fold and seven-fold higher than patients with PsO and RA, respectively. Notably, our systematic search identified two RA datasets where gene expression was assessed through synovial biopsies, while eight datasets were based on peripheral blood samples. Notwithstanding the importance of whole blood gene expression analyses, the invasive procedure of synovial biopsies would be able to identify the disease-specific deregulated genes and pathways that are involved in the arthritic inflammation [66]. Furthermore, the heterogeneity of each specific dataset, due to the different experimental procedures performed, alongside the systematic differences present in every high-throughput experiment, introduces batch effects, impairing the analytical procedures conducted in a meta-analysis. Nevertheless, our late-stage integration approach is able to eliminate, to a significant extent, such batch effects without removing the between-samples biological heterogeneity through the objective meta-analysis of the summary statistics of each separate dataset. Additionally, biological heterogeneity is not exclusively due to the response to therapy; the incorporation of clinical variables that are associated with the clinical outcome, as well as the quality and amount of the isolated RNA, is crucial in understanding the divergence of such complex phenotypes.

Despite the limitations present in every meta-analysis of high-throughput experiments, our analyses provide a framework for the objective distinguishing of deregulated genes and pathways during the response to anti-TNFα therapy. Due to the high cost of the performed experiments, a late-stage integration of transcriptomic datasets from smaller cohorts is able to further enhance the statistical power of expression signatures. In this way, our results could further serve as an important resource for the clarification of the implicated molecular and immune mechanisms in the response to treatment and, at the same time, providing a systematic appraisal of the expression of potential biomarkers. Clinical variation concerning the response to therapy is still poorly characterized; transcriptomic changes, caused by the interactions of genetic and environmental factors, are able to elucidate the actual mechanisms that lead to the differentiation of responsiveness, thus enabling the identification of unique and shared biomarkers between the three diseases under study.

## 5. Conclusions

In conclusion, the prediction of a patient’s response to biological treatments requires the identification of essential biological pathways and regulatory factors that are implicated in the immune and histological background of each disease. The difficult task of unraveling the complex interactions implicated in the response to anti-TNFα therapy in patients with PsO, IBD, and RA, as well as distinguishing gene clusters and pathways that are altered by this heterogeneous phenotype, could be aided by meta-analysis of such gene expression data.

## Figures and Tables

**Table 1:** Summary of the datasets included in our study.

GSE Series Accession Number	Array Platform	Biopsy	Clinical Assessment	Patients (R/NR)	Treatment
Inflammatory Bowel Disease					
GSE52746 [22]	GPL17996	Intestinal Mucosa	CDEIS	12 (7/5)	ADA, IFX
GSE16879 [23]	GPL570	Intestinal Mucosa	Mayo scores/ Endoscopic healing	60 (27/33)	IFX
GSE92415	GPL13158	Intestinal Mucosa	Mayo scores	50 (29/21)	GOL
GSE23597 [24]	GPL570	Intestinal Mucosa	Mayo scores	29 (16/13)	IFX
Psoriasis					
GSE106992 [25]	GPL570	Skin	PASI	21 (19/2)	ETA
GSE11903 [26]	GPL571	Skin	PASI	15 (11/4)	ETA, ADA
GSE85034 [27]	GPL10558	Skin	PASI	15 (10/5)	ETA, ADA
Rheumatoid Arthritis					
GSE140036 [28]	GPL8234	Synovial	EULAR	11 (8/3)	ADA, IFX, ETA
GSE15602 [29]	GPL570	Synovial	EULAR	11 (8/3)	ADA

Abbreviations: PASI, Psoriasis Area Severity Index; ETA, Etanercept; ADA, Adalimumab; EULAR, European League Against Rheumatism; IFX, Infliximab; CDEIS, Crohn’s Disease Endoscopic Index of Severity; GOL, Golimumab.

**Table 2:** Top five DEGs for each meta-analysis conducted in our study, as derived from the *TopConfects* [39] approach.

Disease	Symbol	log_2_(FC)	*p* Value	Disease	Symbol	log_2_(FC)	*p* Value
Down-regulated				Up-regulated			
IBD	*PROK2*	−2.36027	2.93 × 10^−20^	IBD	*PCK1*	2.129965	1.13 × 10^−10^
	*CHI3L1*	−2.78866	2.05 × 10^−12^		*HMGCS2*	1.998756	2.42 × 10^−11^
	*FCGR3B*	−2.54556	7.94 × 10^−12^		*GUCA2B*	1.778594	1.07 × 10^−12^
	*S100A12*	−1.94699	6.70 × 10^−16^		*GUCA1B*	1.683352	3.74 × 10^−13^
	*FPR1*	−1.80196	7.42 × 10^−17^		*TRPM6*	1.657438	1.36 × 10^−12^
Psoriasis	*PRDM1*	−0.62185	7.66 × 10^−7^	Psoriasis	*PHF1*	0.501467	4.43 × 10^−7^
	*ABHD17C*	−0.54337	5.58 × 10^−6^		*C1orf115*	0.646972	1.61 × 10^−6^
	*SERPINB13*	−0.8009	8.94 × 10^−6^		*DEPP1*	0.720268	1.87 × 10^−5^
	*THBD*	−0.51355	6.97 × 10^−6^		*MIR7114*	0.48793	1.45 × 10^−5^
	*DNAJB6*	−0.39466	4.72 × 10^−6^		*ASMTL*	0.420074	2.19 × 10^−5^
RA	*C1QTNF6*	−1.14956	6.59 × 10^−7^	RA	*AK1*	1.206349	2.48 × 10^−6^
	*FMO1*	−1.93396	4.47 × 10^−6^		*NAV1*	0.68339	2.54 × 10^−7^
	*IL2RA*	−1.16699	3.35 × 10^−6^		*PLAC9*	0.886543	2.40 × 10^−6^
	*IL12RB2*	−0.50277	7.88 × 10^−8^		*SOX8*	1.324758	1.90 × 10^−5^
	*SIGLEC7*	−0.56548	1.73 × 10^−6^		*GPSM2*	0.908652	2.87 × 10^−5^
Combined	*S100A9*	−1.7893	1.88 × 10^−10^	Combined	*ZNF91*	0.670157	1.13 × 10^−13^
	*GNA15*	−0.64899	6.11 × 10^−14^		*TRIM2*	0.518893	2.70 × 10^−12^
	*NOD2*	−0.63377	1.29 × 10^−13^		*NR3C2*	0.515162	1.80 × 10^−11^
	*PRDM1*	−0.60289	5.49 × 10^−12^		*TCEA3*	0.549032	6.47 × 10^−09^
	*LYN*	−0.54475	2.33 × 10^−13^		*PHYH*	0.410147	2.68 × 10^−10^

## Data Availability

All, raw and processed from the corresponding authors, datasets included in our study are available from the Gene Expression Omnibus database repository (https://www.ncbi.nlm.nih.gov/geo/, accessed on 10 November 2021).

## Supplementary Materials

The following supporting information can be downloaded at: https://www.mdpi.com/article/10.3390/genes13050776/s1, Supplementary File S1: Crohn’s disease and Ulcerative Colitis meta-analyses; Supplementary Figure S1: Volcano Plots of Differentially Expressed Genes in IBD, PsO, RA and Combined meta-analysis; Supplementary Figure S2: Density plot of the Heterogeneity P values as derived from our disease-specific meta-analyses; Supplementary Figure S3: Density plot of the Heterogeneity P values as derived from our combined meta-analysis; Supplementary Figure S4: Density plot of the Heterogeneity P values as derived from our Crohn’s disease and Ulcerative colitis meta-analyses; Supplementary Figure S5: Volcano Plots of Differentially Expressed Genes in CD and UC; Supplementary Figure S6: Comparison of the enriched Reactome pathways for the down-regulated Crohn’s disease and Ulcerative Colitis genes; Supplementary Figure S7: Semantic similarity analysis of the simplified Biological Processes considering the down-regulated genes in Crohn’s disease; Supplementary Figure S8: Semantic similarity analysis of the simplified Biological Processes considering the down-regulated genes in Ulcerative Colitis; Supplementary Table S1: Differentially expressed genes across all our meta-analyses; Supplementary Table S2: List of all enriched Reactome pathways terms across all our meta-analyses; Supplementary Table S3: List of all enriched Gene Ontology terms across all our meta-analyses.
