# Supplementary material for: Gene Expression Meta-Analysis of Potential Shared and Unique Pathways between Autoimmune Diseases under Anti-TNFα Therapy
Source: Genes (Basel). 2022 Apr 27;13(5):776. doi: 10.3390/genes13050776 (PMC9140437; doi:10.3390/genes13050776)
Supplement: Supplementary file 1 [file genes-13-00776-s001.zip › Supplementary Figures.pdf]

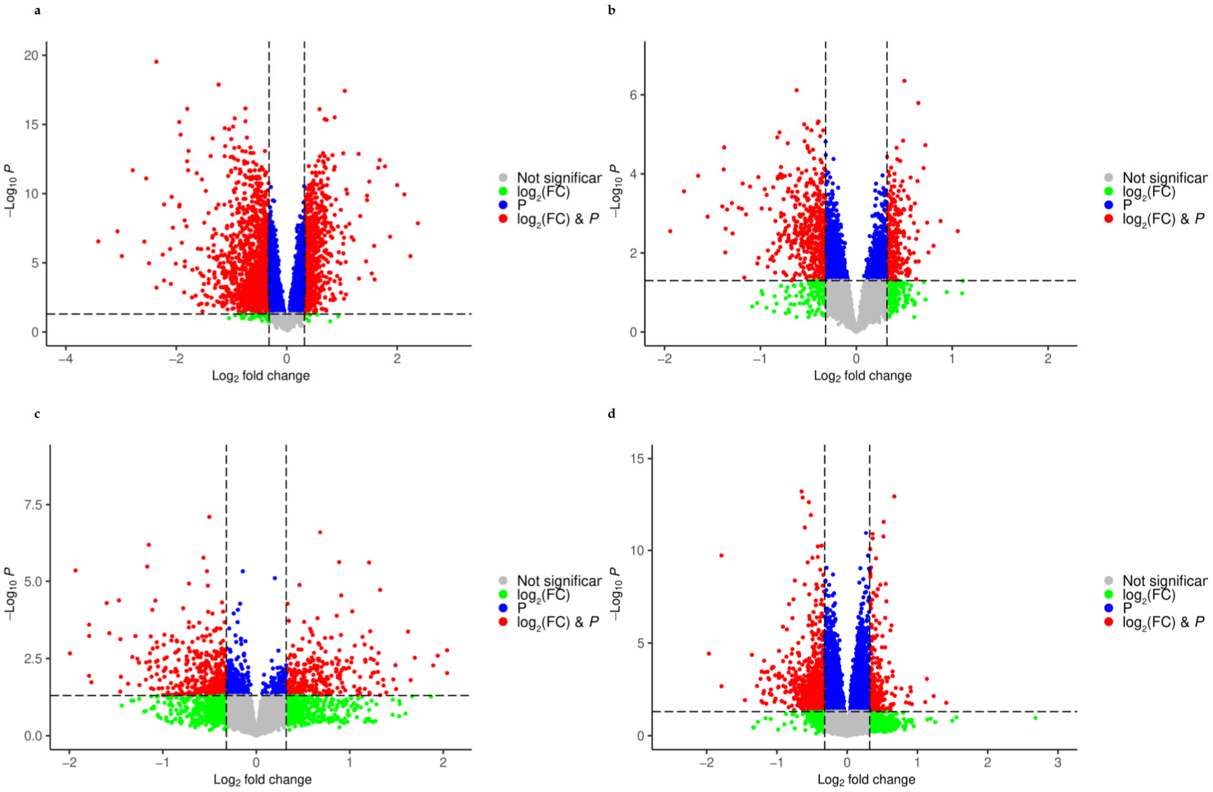

Supplementary Figure S1. Volcano Plots of Differentially Expressed Genes in (**a**) IBD, (**b**) PsO, (**c**) RA and (**d**) Combined meta-analysis. Gray plots represent non-significant genes, blue dots represent genes that met the P criteria, green dots represent genes that met the logFC threshold criteria, while red plots represent genes that met both logFC and P criteria.

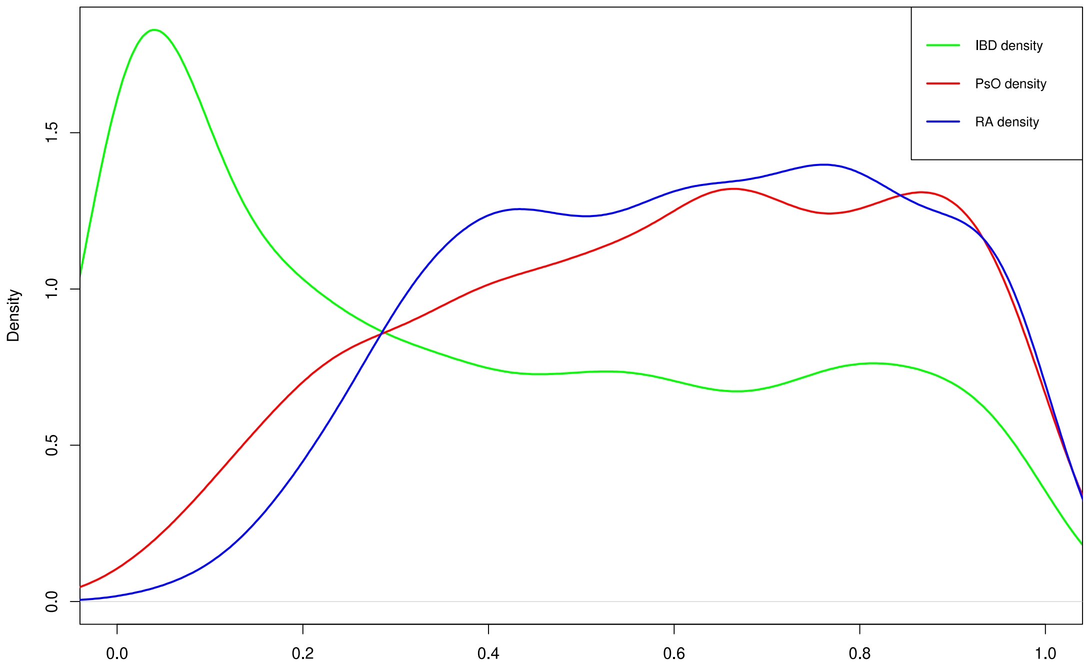

Supplementary Figure S2. Density plot of the Heterogeneity *P* values as derived from our disease-specific meta-analyses.

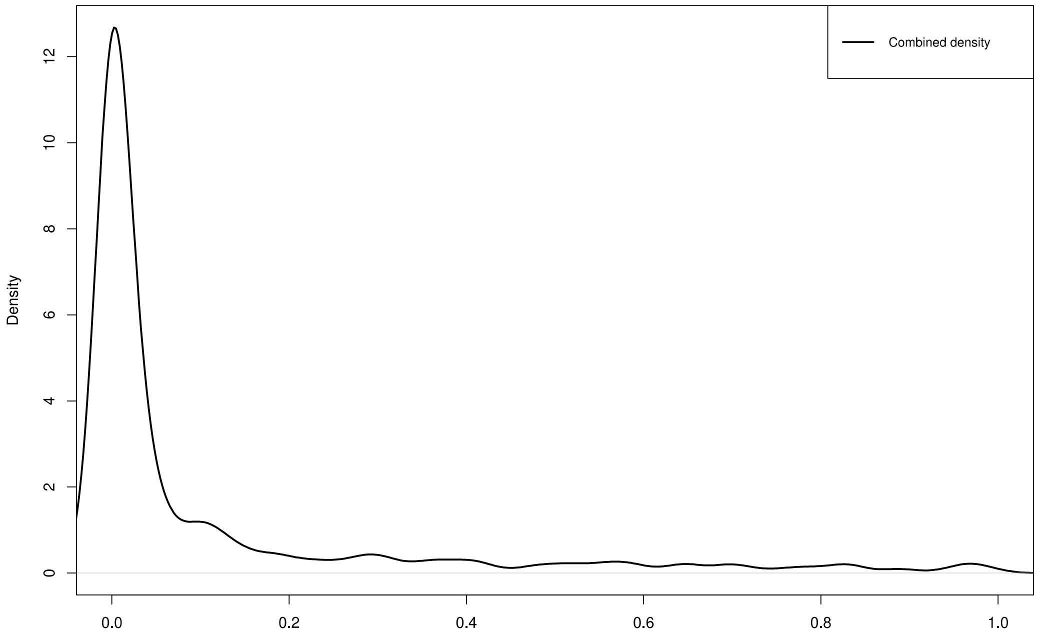

Supplementary Figure S3. Density plot of the Heterogeneity *P* values as derived from our combined meta-analysis.

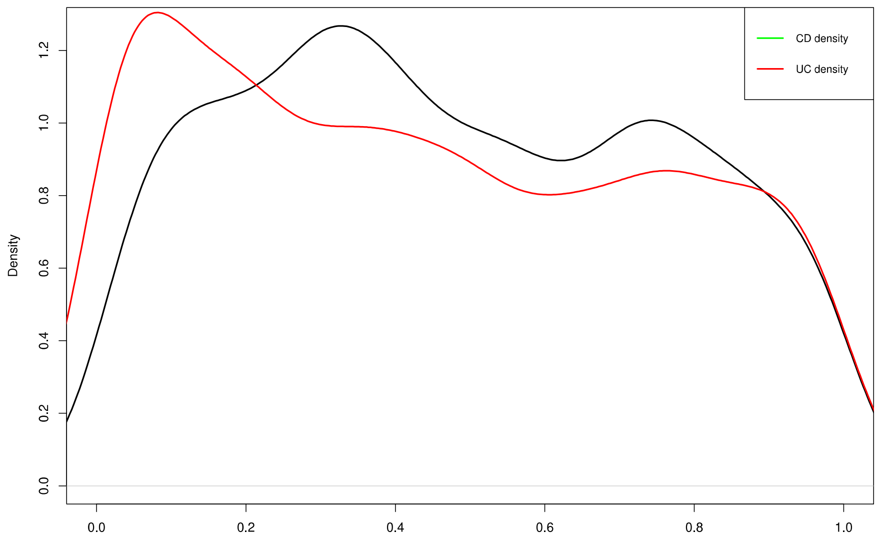

Supplementary Figure S4. Density plot of the Heterogeneity *P* values as derived from our Crohn's disease and Ulcerative colitis meta-analyses.

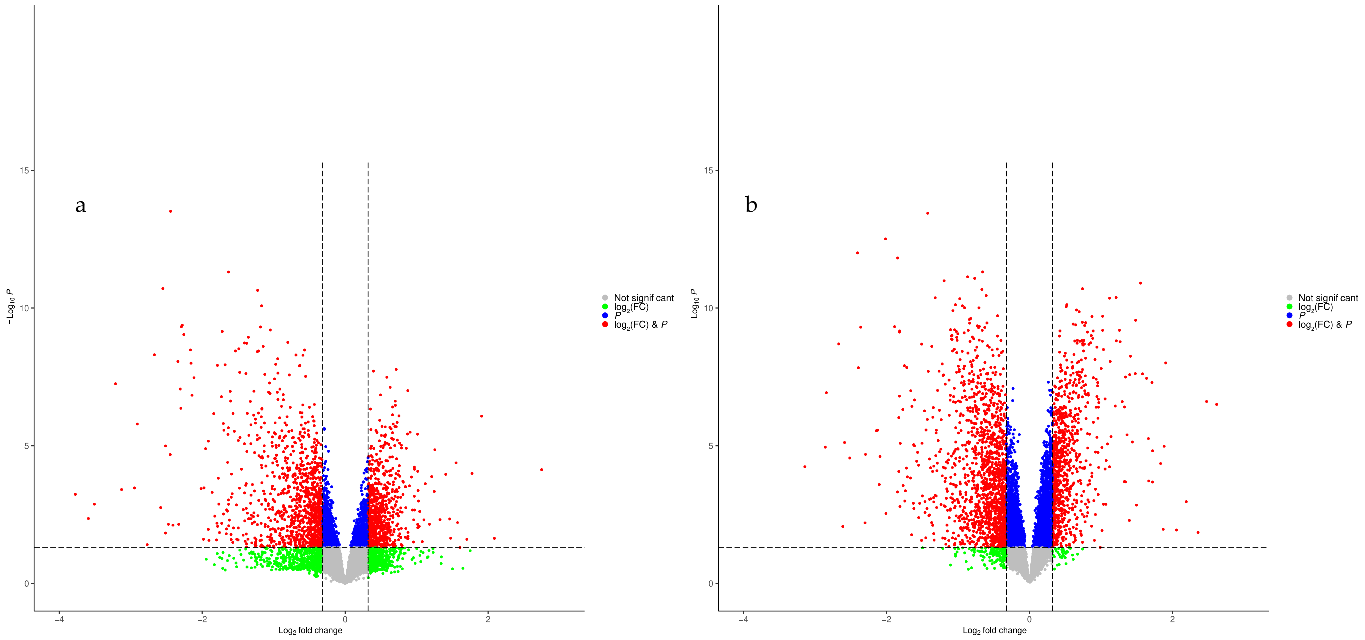

Supplementary Figure S5. Volcano Plots of Differentially Expressed Genes in (**a**) CD and (**b**) UC. Gray plots represent non-significant genes, blue dots represent genes that met the P criteria, green dots represent genes that met the logFC threshold criteria, while red plots represent genes that met both logFC and P criteria.

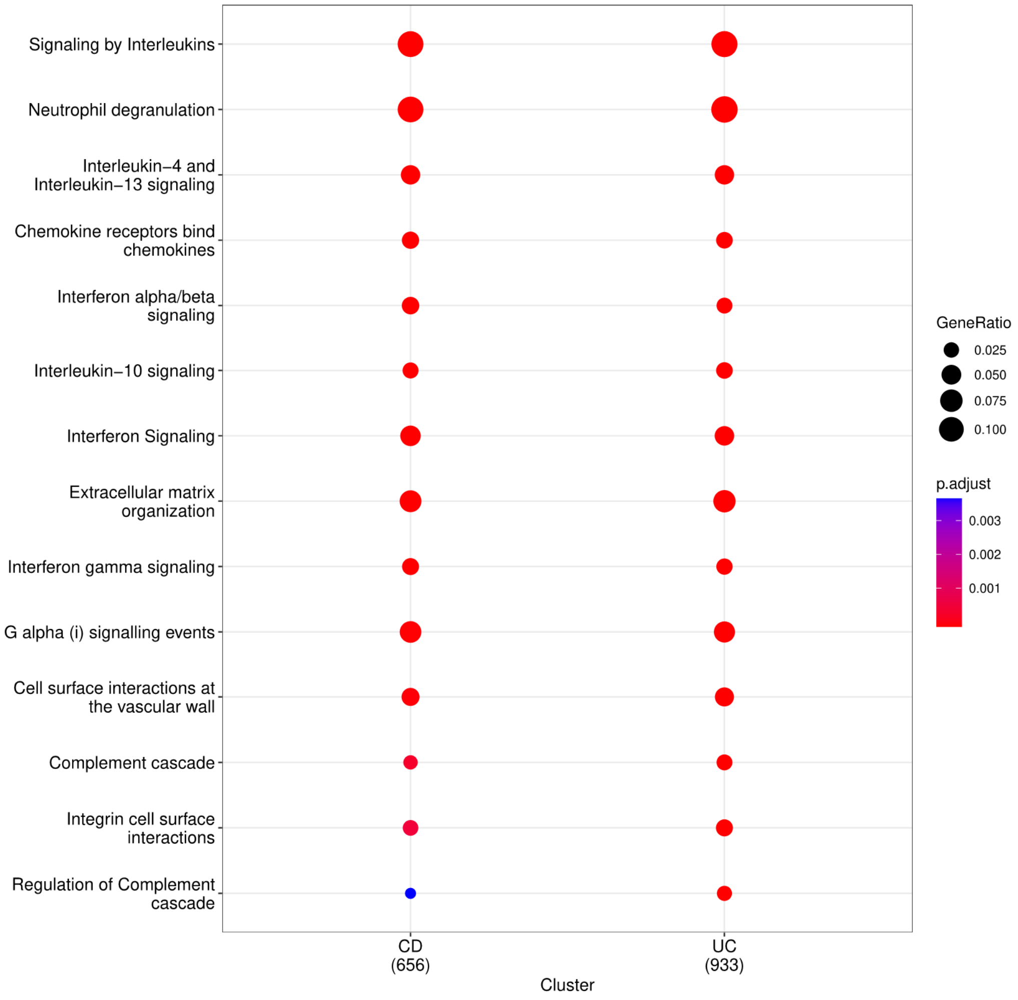

Supplementary Figure S6. Comparison of the enriched Reactome pathways for the down-regulated Crohn's disease and Ulcerative Colitis genes.

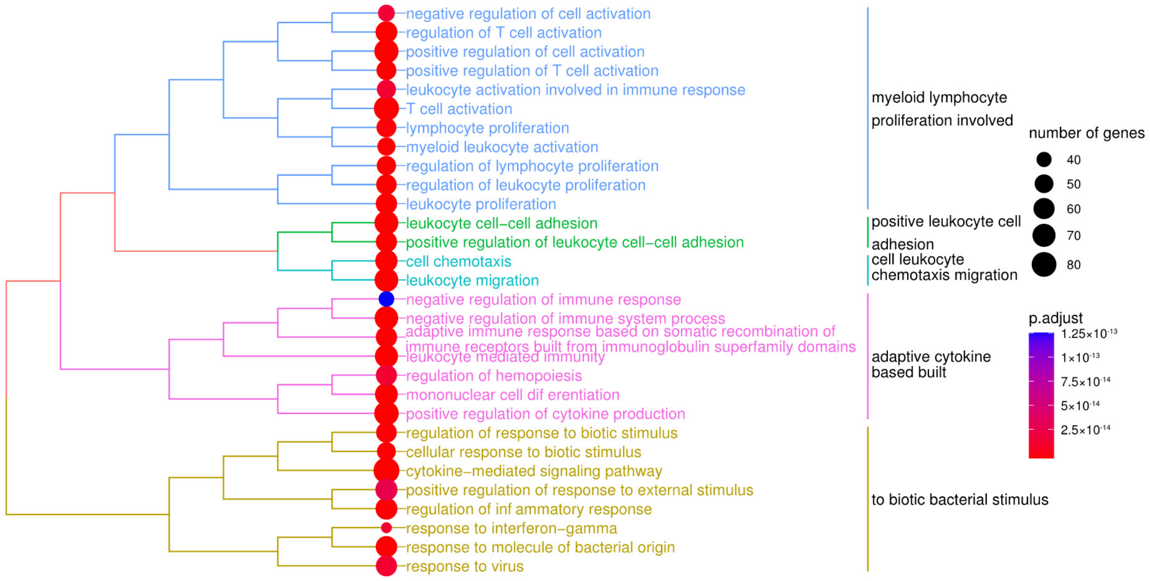

Supplementary Figure S7. Semantic similarity analysis of the simplified Biological Processes considering the down-regulated genes in Crohn's disease.

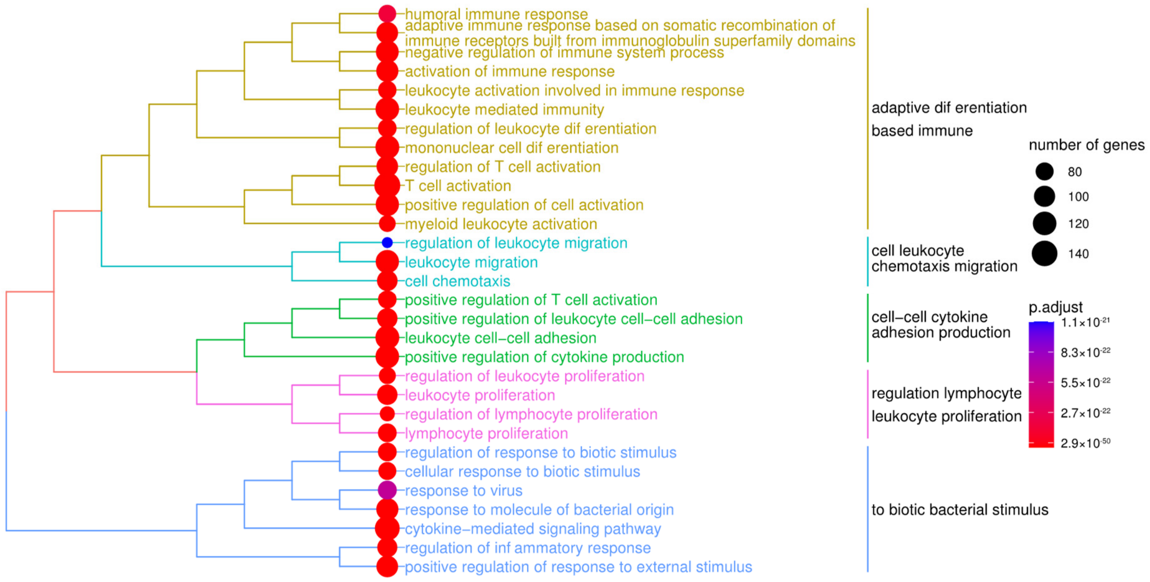

Supplementary Figure S8. Semantic similarity analysis of the simplified Biological Processes considering the down-regulated genes in Ulcerative Colitis.
