# Supplementary material for: Gene Expression Meta-Analysis of Potential Shared and Unique Pathways between Autoimmune Diseases under Anti-TNFα Therapy
Source: Genes (Basel). 2022 Apr 27;13(5):776. doi: 10.3390/genes13050776 (PMC9140437; doi:10.3390/genes13050776)
Supplement: Supplementary file 1 [file genes-13-00776-s001.zip › Supplementary File S1-edit.pdf]

Crohn's Disease and Ulcerative Colitis sub meta-analyses

Materials and Methods

After our systematic literature search and application of our inclusion/exclusion criteria, we identified four inflammatory bowel disease (IBD)-related microarray datasets. Out of those, GSE52746 referred to Crohn's disease (CD) patients, GSE92415 and GSE23597 datasets referred to only ulcerative colitis (UC) patients, and GSE16879 studies both CD and UC patients. Although the study of UC and CD under the IBD term is commonly utilized, the distinct etiopathological mechanisms that lead to the development of each disease are different. Consequently, we conducted meta-analysis between responders and non-responders to anti-TNFα therapy in patients with Crohn's disease and ulcerative colitis, as derived from our included datasets. Our methodology for these sub-analyses was common with our main methodology, as presented in the main text. Briefly, differential gene expression analysis (DGEA) was performed through the *limma* [37] package for each separate dataset, where fold change and 95% confidence intervals (95% CI) were calculated and utilized for our random effects meta-analysis implicated in the *MetaVolcanoR* package [38]. The top 1% of the most consistently perturbed genes in the included datasets were highlighted while maintaining a 5% false discovery rate (FDR) through the TopConfects approach [39]. Genes were considered differentially expressed (DEGs) with $P \leq 0.05$, $|\log_2(FC)| \geq \log_2(1.25)$ and perturbed in at least 75% of the included datasets.

Over-representation analysis (ORA) was performed using gene ontology (GO) [41] and reactome pathways [42] with the R *clusterProfiler* package [43] for both up- and down-regulated genes in UC and CD meta-analyses using the default parameters. Outputs of the GO ORA were further analyzed and reduced to single representative terms to reduce the redundancy of the GO child terms. Pairwise similarities were subsequently calculated for the biological processes (BPs) of each down-regulated gene set through Wang's method based on the topology of GO-directed acyclic graphs (DAGs) [44]. For each enriched pathway, the *P* value was calculated with the hypergeometric model and controlled for multiple comparisons with the Benjamini and Hochberg method. Enriched pathways with adjusted $P \leq 0.05$ were regarded as statistically significant.

Results

We identified four IBD datasets. Out of those, two (GSE52746, GSE16879) included patients with CD. Twelve (seven responders/five non-responders) patients with CD were identified from GSE52746, while 36 (20 responders/16 non-responders) CD patients were included in GSE16879. Considering the UC patients,

24 patients (7 responders/17 non-responders) were included in GSE16879, 50 (29 responders/21 non-responders) in GSE92415 and 29 (16 responders/13 non-responders) in GSE23597 (Table 3). The clinical characterization of the response to therapy was evenly distributed amongst UC datasets, where decrease to Mayo endoscopic subscores of 0 or 1 considered patients as responders [23]. On the other hand, patients in the GSE52746 dataset were considered responders by the decrease in the Crohn's disease Index of Severity (CDEIS) index of less than 5, while responders in the GSE16879 dataset should have a complete mucosal healing with a significant decrease at the histological score [48].

Table 3. Summary of the datasets included in our study.

GSE Series Accession Number	Array Platform	Biopsy	Clinical Assessment	Patients (R/NR)	Treatment
Crohn's Disease					
GSE52746 [22]	GPL17996	Intestinal Mucosa	CDEIS	12 (7/5)	ADA, IFX
GSE16879 [23]	GPL570	Intestinal Mucosa	Endoscopic healing	36 (20/16)	IFX
Ulcerative Colitis					
GSE16879 [23]	GPL570	Intestinal Mucosa	Mayo scores	24 (7/17)	IFX
GSE92415	GPL13158	Intestinal Mucosa	Mayo scores	50 (29/21)	GOL
GSE23597 [24]	GPL570	Intestinal Mucosa	Mayo scores	29 (16/13)	IFX

Abbreviations: ADA, Adalimumab; IFX, Infliximab; CDEIS, Crohn's Disease Index of Severity; GOL, Golimumab

The CD meta-analysis yielded a total of 1,798 differentially expressed genes (DEGs), where 757 were up-regulated and 1,041 were down-regulated. On the other hand, UC meta-analysis gave us a total of 2,297 DEGs, out of which 880 were up- and 1,417 were down-regulated (Supplementary Figure 5). Overall, 1066 DEGs were shared between UC and CD meta-analysis, with 685 being down- and 381 being up-regulated. A full list of our DEGs considering both meta-analyses is provided at Supplementary Table 1.

ORA performed for each meta-analysis on the Reactome database pathways showed 47 statistically significant enriched pathways for the down-regulated CD genes and 94 for the down-regulated UC genes. Interestingly, 41 pathways were shared between both diseases, which mostly referred to immune pathways, such as Interleukin Signaling and Extracellular matrix organization (Supplementary Figure 6). However, when considering the up-regulated genes, enriched pathways showed vast disparities between UC and CD. CD up-regulated genes were enriched for multiple metabolic pathways, such as citric acid (TCA) cycle (15/55 genes) and phospholipid metabolic pathways (23/212 genes), while UC up-regulated genes revealed transport activities (Ion channel transport, 21/186 genes), metal-related pathways (Metallothioneins bind metals, 6/11 genes; response to metal ions, 6/14 genes), as well as metabolic pathways (Digestion, 6/22 genes) (Supplementary Table 2). Considering the GO enrichment analysis, 935

BP terms were enriched considering the down-regulated CD genes, which were further simplified to 291 distinct terms, while UC BP terms were reduced from 1,424 to 397 (Supplementary File 4). Similar to the reactome enrichment analysis, as seen through our semantic similarity analysis presented in Supplementary Figures 7 and 8, both CD and UC terms referred to similar terms such as response to bacterial infections, chemotaxis and activation of T cells. Up-regulated CD genes, however, showed us 61 simplified BP terms related to metabolic processes of pyruvate, fatty acids, and alcohol, as well as transporting activities. On the other hand, UC up-regulated genes were enriched for 98 simplified BP terms which referred to catabolic processes of organic acids, lipids, and transporting activities of metal ions (Supplementary Table 3).
